# Household Expenditure for Dental Care in Low and Middle Income Countries

**DOI:** 10.1371/journal.pone.0123075

**Published:** 2015-04-29

**Authors:** Mohd Masood, Aubrey Sheiham, Eduardo Bernabé

**Affiliations:** 1 Centre of Population Oral Health & Clinical Prevention Studies, Faculty of Dentistry, Universiti Teknologi MARA, Shah Alam, Malaysia; 2 Department of Epidemiology and Public Health, University College London, London, United Kingdom; 3 Division of Population and Patient Health, King’s College London Dental Institute at Guy’s, King’s College and St. Thomas’ Hospitals, London, United Kingdom; University of Washington, UNITED STATES

## Abstract

This study assessed the extent of household catastrophic expenditure in dental health care and its possible determinants in 41 low and middle income countries. Data from 182,007 respondents aged 18 years and over (69,315 in 18 low income countries, 59,645 in 15 lower middle income countries and 53,047 in 8 upper middle income countries) who participated in the WHO World Health Survey (WHS) were analyzed. Expenditure in dental health care was defined as catastrophic if it was equal to or higher than 40% of the household capacity to pay. A number of individual and country-level factors were assessed as potential determinants of catastrophic dental health expenditure (CDHE) in multilevel logistic regression with individuals nested within countries. Up to 7% of households in low and middle income countries faced CDHE in the last 4 weeks. This proportion rose up to 35% among households that incurred some dental health expenditure within the same period. The multilevel model showed that wealthier, urban and larger households and more economically developed countries had higher odds of facing CDHE. The results of this study show that payments for dental health care can be a considerable burden on households, to the extent of preventing expenditure on basic necessities. They also help characterize households more likely to incur catastrophic expenditure on dental health care. Alternative health care financing strategies and policies targeted to improve fairness in financial contribution are urgently required in low and middle income countries.

## Introduction

The financial burden of illness and out-of-pocket expenditure on health care has been the focus of increasing attention in recent years. Out-of-pocket payments are the primary source of health care financing in many countries, particularly in the developing world [1], and are considered ‘catastrophic’ when they force households into having to reduce expenditure on basic necessities [2,3]. The share of health expenditure, or the percentage of the household expenditure spent on health care, is used to determine the number of households incurring catastrophic health expenditures (CHE) and to derive estimates of private or out-of-pocket health expenditure reported in national health accounts [4,5].

Few studies have evaluated CHE across countries, with varied methodologies [2,3,6–8]. In the largest study to date including 89 countries covering 89% of the world population, 3% of households in low income countries, 1.8% of households in middle income countries and 0.6% of households in high income countries incur CHE [3]. Countries with lower Gross Domestic Product (GDP) per capita and greater income inequality were more likely to have higher proportions of households facing CHE [3] whereas rural residence, low income, presence of older adults and/or young children and lack of health insurance in the household were associated with higher propensity of CHE [2,8].

Treating oral diseases is costly [9], even In high income countries where 5–10% of public health spending is used for dental care [10]. Although there is no equivalent data for low income countries, it has been estimated that treating caries in children would cost between US$ 1618 and 3513 per 1000 children of mixed ages from 6 to 18 years, an amount that exceeds the available resources for the provision of an essential public health care package for the children of most low income countries [11]. Those needing dental treatment face two important economic consequences: the high direct costs of the service (out-of-pocket expenditure) and the indirect loss of income and productivity to attend services [12]. Using dental services can cost households a large proportion of their available income and push many into poverty and long-term debt. However, the burden of out-of-pocket payments for dental care is not well documented in the literature despite the fact this information represents the failure of the health system to protect the public from the financial consequences of health care [13] and may pave the way for alternative mechanisms to finance health care provision.

A trend analysis in Mexico showed that 8.5%, 4% and 5% of households had some dental care expenditure during the past 3 months in 2000, 2002 and 2004, respectively, while 0.8%, 0.01% and 1.8% of households incurred catastrophic expenditure because of dental health care in 2000, 2002 and 2004, respectively [14]. Household expenditure in dental care, as a proportion of the household capacity to pay, increased steadily from the highest to the lowest wealth group in all 3 years [14]. Another study in Iran showed that dental health expenditure was a key contributor to CHE. Households using dental services had four times greater odds of facing CHE than those not using such services [15]. The study also found that the unequal utilization of dental health services reduced the inequality in CHE between socioeconomic groups as wealthier households were more likely to incur dental health expenditures [15].

As costs of dental services are high in most countries [9,10] and dental diseases are very common worldwide [16–18], this study aimed to explore the extent of household catastrophic dental health expenditure and its possible determinants in 41 low and middle income countries.

## Methods

### Data source

Data were from the World Health Survey (WHS) conducted in 2002–2004, which was launched by the World Health Organization (WHO) to provide valid, reliable and comparable information across 70 countries from all world regions regarding health status and health systems. The WHS data has been used frequently for the purpose of descriptive and analytical epidemiological investigations.

In each country, the target population was adults aged 18 years and over living in private households. Participants were selected using multistage stratified cluster sampling with the intention of collecting nationally representative samples. However, in six countries (India, China, Comoros, Congo, Ivory Coast and the Russian Federation) the survey was carried out in geographically delimited regions and random sampling was not used. Sample sizes varied between 1000 and 10,000 between different countries while ensuring the sample was nationally representative of the population. In each household, one adult was randomly selected (Kish table) after completing a full household roster [19].

Fifty of the 70 countries that participated in the WHS were classified as low and middle income economies according to World Bank’s classification [20], and were initially selected for this analysis. We excluded the following countries: Guatemala and Zambia because their data files have no survey information (sampling units, stratification or population weights) necessary to produce population-level estimates, Hungary and Turkey because they used a limited set of the questions on household expenditure, and Ecuador, Nepal, Malawi, Slovak Republic and Sri Lanka because they had missing values in more than 60% of the items on household expenditure.

### Variable selection

Expenditure data were collected at household level from the chosen household informant. Respondents were asked to provide information on total household expenditure over the last 4 weeks, and then details of item-by-item expenditure over the same period. The specified items were food, housing, education, health care, voluntary health insurance premiums, and all other goods and services. Respondents were asked to report on both cash and in-kind payments. Eight more detailed questions on health expenditure followed, to elicit information on payments for outpatient services, hospitalization, traditional medicine services, dentists, medication, medical tests, health-care products and other expenditures. Health expenditure excluded cost of transportation to obtain care and was net of insurance reimbursement. Dental health expenditure was defined as ‘catastrophic’ (CDHE) if it was equal to or higher than 40% of the household capacity to pay [2,3]. Although thresholds between 5% and 40% can be used, the highest was used to identify the people facing the most extreme difficulties [2]. The 40% threshold has been adopted by the WHO [21]. Household capacity to pay was calculated as the total household expenditure minus basic subsistence expenditure adjusted for household size [2,3]. Subsistence expenditure—defined as the mean food expenditure of households falling between the 45th and 55th percentiles of the total sample in terms of the share of total household expenditure spent on food—was estimated for each individual country separately [2,3].

A number of individual and country-level factors were included in the analysis as potential determinants of CDHE. Participants’ sex, age, marital status and education as well as household wealth and size were the individual-level factors. Age groups were categorized as 18–29, 30–39, 40–49, 50–59, 60–69 and 70 years and above. Marital status was classified as married (including those cohabiting), never married and previously married (separated, divorced and widowed). Education was measured using a 7-point response scale (no formal schooling, less than primary school, primary school completed, secondary school completed, high school or equivalent completed, college/pre-university/university completed, and postgraduate degree completed) and responses collapsed into three categories (primary school or less, secondary school or college and higher) to enhance comparability across countries. Household wealth was determined using the wealth index, which classifies households based on their ownership of a range of permanent income indicators (household assets) ranging from bicycle, mobile phone, fixed line phones and refrigerator to computer, dish washer, washing machine and car [22,23]. Country-specific items were also added to the list of assets to fit the standard of living of the countries, and the final list included between 11 and 20 items. A principal components analysis (PCA) was then carried out separately for each country to determine the weights to create an index of the asset variables. The weights for the first component were then applied to each person’s data giving a continuous asset index measure [22,24]. This index was then categorized into tertiles. Household size was measured as the number of adults and children in the family. Country-level factors were average national income and income inequality. National income was measured as GDP per capita for the year 2003 converted to current US dollars, which was obtained from the World Bank [25]. Income inequality was measured using the Gini coefficient (expressed in percentage value), which was obtained from the World Bank [25]. Reporting dates vary from country to country but were between the period 2001 and 2005, chosen to match as closely as possible the WHS period.

### Data analysis

Survey analytic procedures were used to account for the complex survey design (stratification and clustering) and incorporate sampling weights to generate population-level estimates and standard errors for each specific country. R-3.1.0 for Windows with different packages was used for all the analyses. Survey package was used for all design-based analyses and lme4 package with glmer command was used for multilevel logistic regression analysis.

The full sample achieved by the WHS and our study sample for each country are first presented. CDHE for each country was reported for the full sample of respondents as well as for households who reported any dental health expenditure in the last 4 weeks. Data were presented for low, lower middle and upper middle income countries (LIC, LMIC and UMIC respectively).

A two-level random-intercepts and fixed-slopes model structure with individuals nested within countries was fitted, treating CDHE as a binary outcome and using logistic regression. The fixed- and random-parameter estimates for the two-level logistic regression model were calculated using the adaptive Gauss-Hermite approximation to the log-likelihood, as implemented in R. Multilevel modeling incorporating survey design features is a matter of ongoing debate [26,27] and not currently available in R, therefore results from multilevel modeling were not weighted. Our model strategy was first to estimate the null model (labeled as Model 0) and then to include explanatory variables gradually into the model. All individual-level factors (age, sex, marital status, education, household wealth and size and rural/urban status) were included as explanatory variables in Model 1. Country-level factors (GDP per capita and Gini coefficient) were subsequently included as explanatory variables in Model 2.

## Results

Data were from 182,007 respondents aged 18 years and over living in 41 low and middle income countries and who have complete information on all the variables selected for analysis (69,315 in 18 LIC, 59,645 in 15 LMIC and 53,047 in 8 UMIC). Table 1 shows the total number of participants in the WHS and the sample used for this study in each country.

**Table 1:** Number of adults who participated in the World Heath Survey (full sample) and who were included for this analysis (study sample) in 41 low and middle income countries.

Income group	Country	Full sample	Study sample	%
Low Income Countries	Bangladesh	5942	5912	99.5
	Burkina Faso	4945	4570	92.4
	Chad	4866	2928	60.2
	Comoros	1835	1724	94.0
	Congo, Republic	3070	1446	47.1
	Ethiopia	5090	3435	67.5
	Ghana	4159	3070	73.8
	India	10683	6053	56.7
	Ivory Coast	3245	2701	83.2
	Kenya	4639	4012	86.5
	Lao PDR	4989	4939	99.0
	Malawi	5545	5374	96.9
	Mauritania	3844	2794	72.7
	Myanmar	6045	6045	100.0
	Pakistan	6502	5991	92.1
	Senegal	3458	1805	52.2
	Vietnam	4174	3019	72.3
	Zimbabwe	4264	3497	82.0
Lower Middle Income Countries	Bosnia and Herzegovina	1031	769	74.6
	Brazil	5000	4575	91.5
	China	3994	3807	95.3
	Dominican Republic	5027	4596	91.4
	Georgia	2947	2732	92.7
	Kazakhstan	4499	4388	97.5
	Morocco	4716	4450	94.4
	Namibia	4377	3895	89.0
	Paraguay	5288	5215	98.6
	Philippines	10083	9870	97.9
	Russian Federation	4426	4375	98.8
	South Africa	2601	2424	93.2
	Swaziland	3070	2687	87.5
	Tunisia	5199	4365	84.0
	Ukraine	2814	1497	53.2
Upper Middle Income Countries	Croatia	993	894	90.0
	Czech Republic	949	618	65.1
	Estonia	1021	1001	98.0
	Latvia	929	751	80.8
	Malaysia	6145	5355	87.1
	Mauritius	3968	3794	95.6
	Mexico	38746	37669	97.2
	Uruguay	2989	2965	99.2

The proportion of households incurring CDHE in the last 4 weeks ranged from 0.1% in Namibia and Lao to 6.8% in Ukraine. CDHE was more common in more developed countries. Two LIC (11%), 6 LIMC (40%) and 4 UMIC (50%) had at least 1% of households facing CDHE. To capture the impact of CDHE among households that incurred dental health expenditures in the last 4 weeks, a separate analysis was conducted excluding households with no dental spending. This figure represents households whose expenditure on dental care in the last 4 weeks was catastrophic. Percentages ranged from 2.8% in Swaziland to 35.0% in Ukraine. CDHE was more common in more developed countries; 9 LIC (50%), 9 LIMC (60%) and 6 UMIC (75%) had at least 10% of households whose expenditure on dental care was catastrophic (Table 2).

**Table 2:** Catastrophic dental health expenditure (CDHE) for 41 low middle income countries.

Income group	Country	CDHE as % of full study sample (95% CI)		CDHE as % of those with DHE>0 (95% CI)	
Low Income Countries	Bangladesh	0.7	(0.5–1.0)	8.6	(5.9–11.9)
	Burkina Faso	0.2	(0.1–0.3)	10.2	(4.5–18.6)
	Chad	0.6	(0.4–1.0)	18.6	(10.7–28.6)
	Comoros	0.9	(0.5–1.6)	9.4	(5.0–15.6)
	Congo, Republic	1.9	(0.3–6.0)	30.1	(6.8–64.7)
	Ethiopia	0.3	(0.1–0.6)	16.1	(6.8–29.9)
	Ghana	0.3	(0.1–0.5)	11.5	(5.1–21.1)
	India	0.6	(0.4–1.0)	8.7	(5.4–12.9)
	Ivory Coast	0.5	(0.3–0.9)	13.3	(6.9–22.1)
	Kenya	0.4	(0.2–0.8)	8.3	(3.2–16.6)
	Lao PDR	0.1	(0.1–0.3)	7.5	(2.8–15.2)
	Malawi	0.2	(0.1–0.3)	9.0	(2.7–20.2)
	Mauritania	1.3	(0.8–2.1)	17.7	(11.3–25.6)
	Myanmar	0.2	(0.1–0.3)	10.3	(4.3–19.6)
	Pakistan	0.5	(0.3–0.8)	4.9	(2.7–7.9)
	Senegal	0.6	(0.3–1.0)	4.9	(2.4–8.7)
	Vietnam	0.3	(0.1–0.8)	14.2	(5.4–28.0)
	Zimbabwe	0.3	(0.1–0.7)	9.7	(3.5–19.9)
Lower Middle Income Countries	Bosnia and Herzegovina	0.8	(0.2–2.1)	5.7	(1.0–16.3)
	Brazil	3.3	(2.8–4.0)	25.3	(21.2–29.6)
	China	0.3	(0.1–0.7)	13.3	(5.5–25.1)
	Dominican Republic	0.9	(0.6–1.3)	15.9	(10.3–22.8)
	Georgia	1.7	(1.2–2.4)	14.1	(8.9–20.7)
	Kazakhstan	1.0	(0.6–1.6)	9.6	(6.3–13.9)
	Morocco	0.9	(0.5–1.5)	10.6	(5.9–17.1)
	Namibia	0.1	(0.0–0.3)	4.4	(1.1–11.4)
	Paraguay	2.1	(1.6–2.6)	16.6	(13.2–20.4)
	Philippines	0.6	(0.4–0.9)	11.9	(7.7–17.3)
	Russian Federation	1.8	(0.7–3.8)	7.6	(3.0–15.1)
	South Africa	0.4	(0.2–0.8)	9.1	(4.0–16.9)
	Swaziland	0.3	(0.1–0.6)	2.8	(1.0–6.0)
	Tunisia	1.1	(0.7–1.7)	21.5	(14.5–29.7)
	Ukraine	6.8	(3.0–12.8)	35.0	(18.8–54.0)
Upper Middle Income Countries	Croatia	0.9	(0.4–1.8)	12.2	(4.6–24.3)
	Czech Republic	1.1	(0.4–2.4)	11.5	(3.1–26.9)
	Estonia	1.0	(0.4–1.9)	15.4	(10.7–21.0)
	Latvia	2.2	(1.2–3.5)	18.2	(10.5–28.0)
	Malaysia	0.4	(0.3–0.7)	7.2	(4.6–10.5)
	Mauritius	0.7	(0.4–1.1)	12.6	(7.4–19.4)
	Mexico	3.5	(3.2–3.9)	31.0	(28.7–33.5)
	Uruguay	0.7	(0.4–0.9)	6.8	(4.9–9.0)

Table 3 presents the results from the multilevel logistic regression analysis. Only 17.5% of total variation in CDHE was found at country level. Respondent’s age, education and marital status, household wealth and size, urban/rural status and average national income were significantly related to CDHE. The odds of incurring CDHE increased with age but were not significant for 60–69 and 70+ year olds compared to 18-29-year-olds. Adults with secondary school (1.36, 95% CI: 1.16–1.59) and college or above education (1.45, 95% CI: 1.15–1.83) had greater odds of incurring CDHE than those with primary school. Previously married adults had lower odds of incurring CDHE (0.84, 95% CI: 0.72–0.98) than married adults. Households in the top wealth tertile had higher odds of incurring CDHE (1.58, 95% CI: 1.38–1.81) than those in the bottom tertile. In terms of household size, families with 3 or more children had lower odds of facing CDHE (0.63, 95% CI: 0.51–0.79) than those with no children whereas families with 3 or more adults had higher odds of facing CDHE than single adult families (1.38, 95% CI: 1.13–1.67). The odds of incurring CDHE were lower for households in rural areas than for those in urban areas (0.82, 95% CI: 0.72–0.93). At country level, the odds of facing CDHE increased 1.17 times (95% CI: 1.06–1.30) for every $US1000 increase in GDP per capita.

**Table 3:** Country- and individual-level factors associated with Catastrophic Dental Health Expenditure (CDHE) among 182,007 adults in 41 low and middle income countries.

	Model 0^a^	Model 1^a^	Model 2^a^
	OR^b^ (95% CI)	OR^b^ (95% CI)	OR^b^ (95% CI)
**Fixed effects: Individual Level**			
*Sex*			
Women		1.00 (Reference)	1.00 (Reference)
Men		0.93 (0.84–1.04)	0.93 (0.84–1.04)
*Age*			
18–29 years		1.00 (Reference)	1.00 (Reference)
30–39 years		1.32 (1.13–1.54) ***	1.31 (1.12–1.54) ***
40–49 years		1.23 (1.03–1.46)*	1.22 (1.03–1.46) *
50–59 years		1.27 (1.05–1.55) *	1.26 (1.04–1.54) *
60–69 years		1.24 (0.99–1.54)	1.23 (0.98–1.53)
70+ years		1.23 (0.96–1.57)	1.22 (0.95–1.57)
*Marital Status*			
Married		1.00 (Reference)	1.00 (Reference)
Never married		0.99 (0.84–1.17)	0.99 (0.84–1.17)
Previously married		0.84 (0.72–0.98) *	0.84 (0.72–0.98) *
*Education*			
Primary school		1.00 (Reference)	1.00 (Reference)
Secondary school		1.38 (1.18–1.62)***	1.36 (1.16–1.59) ***
College and above		1.47 (1.16–1.84)**	1.45 (1.15–1.83) **
*Household wealth*			
First tertile (Poorest)		1.00 (Reference)	1.00 (Reference)
Second tertile (Middle)		1.13 (0.98–1.29)	1.13 (0.98–1.29)
Third tertile (Wealthiest)		1.58 (1.38–1.81) ***	1.58 (1.38–1.81) ***
*Children in household*			
0		1.00 (Reference)	1.00 (Reference)
1–2		1.00 (0.95–1.23)	1.09 (0.96–1.24)
3 or more		0.63 (0.51–0.78) ***	0.63 (0.51–0.79) ***
*Adults in household*			
1		1.00 (Reference)	1.00 (Reference)
2		1.09 (0.91–1.32)	1.09 (0.91–1.31)
3 or more		1.37 (1.12–1.69) **	1.38 (1.13–1.67) **
*Urban/rural status*			
Urban		1.00 (Reference)	1.00 (Reference)
Rural		0.82 (0.72–0.93) **	0.82 (0.72–0.93) **
**Fixed effects: Country Level**			
GDP per capita (1000-increase)			1.17 (1.06–1.30) **
Gini index (1-percent increase)			0.99 (0.96–1.02)
**Random effects**			
Country (SD)	0.70 (0.84)	0.61 (0.78)	0.47 (0.69)

^a^ Model 0 had no explanatory variables (null model), Model 1 adjusted for all individual level factors; and Model 2 also adjusted for country-level factors.

^b^ Two-level logistic regression was fitted and odds ratios (OR) reported.

*<0.05

**<0.01

***<0.001

## Discussion

We found that up to 7% of households in low and middle income countries faced CDHE during the last 4 weeks. That is, the money they spent on dental health care exceeded 40% of income remaining after subsistence needs have been met. The proportion of households facing CDHE was up to 35% among those that incurred some dental spending in the last 4 weeks. CDHE was more common in wealthier, urban and larger households and in more economically developed countries.

Some study limitations need to be kept in mind when interpreting the present results. First, our CDHE estimates were based on 14 questions and a 4-week recall period. It has been previously shown that the magnitude of out-of-pocket and catastrophic spending on health is affected by the number of questions and recall period used to collect data. Estimates of heath spending are higher when using more health expenditure questions and lower when using more non-health expenditure questions and longer recall periods [5,28,29]. Because of these limitations, some have advocated an integrative approach to estimate health expenditure that involves use of all available data sources to triangulate flows of funds from these different channels [5]. Although this approach is ideal, it is not very practical, especially in the short-term for low and middle income countries where few surveys are conducted. More importantly, the WHS expenditure data have been shown to be reliable, based on test-retest estimates [4]. Second, our analysis did not contain data on the indirect costs of seeking dental care, including income loss due to ill health, travel, waiting at health care facilities or providing care to family members [1]. Moreover, our analysis did not allow the assessment of the cumulative effects of oral diseases and recurrent restorative treatment on expenditure on dental health care. Therefore, our estimates of CDHE probably underestimate the financial consequences of out-of-pocket payments for dental health care on households.

Despite this underestimation, our findings show that CDHE is a common problem in low and middle income countries (higher than 1% in 12 of the 41 countries included in this analysis). Our estimates of CDHE are relatively similar compared to those for CHE from previous multi-country studies [2,3,6–8], suggesting that out-of-pocket payments for dental care may be an important contributor to overall CHE as initially found in a study in Iran [15]. In addition, the present results highlight the low level of financial protection that healthcare financing systems provide for their citizens.

Although the same determinants of CHE [2,3,8] were related to CDHE, they had opposite directions. CDHE was more common in wealthier, urban and larger households and in more economically developed countries. Unlike overall health care, dental care in developing countries is financed primarily through out-of-pocket spending, with or without third-party payment schemes [10]. The higher odds of facing CDHE among wealthier and urban households could be because they are more likely to utilize higher cost private providers than lower cost public sector facilities. In addition, the use of dental services in low and middle income countries is not a function of population health needs, but rather the individual household’s ability to pay for those services [14]. Our findings may thus relate to those who have found a way to access and use dental health services. Larger households are more likely to face CDHE because they have more individuals at risk of oral disease, including vulnerable members such as older adults. As for families having children, most developing countries have government-funded health services for children. That reduces the monetary burden on children’s families [30].

At country level, the odds of facing CDHE increased with GDP per capita. Although households’ capacity to pay is related to economic growth [3], CHE is more common in countries with high levels of poverty and health care utilization [2]. Poverty does not only occur in LIC but is high in LMIC and UMIC. On the other hand, the proportion of adults using dental services increases with GDP per capita [31]. It is also possible that teeth and dental appearance play a stronger role in individuals’ integration to society (including social position and work roles) in more developed countries [32].

Our findings have implications for policy and research. They indicate that current mechanisms for financing dental care in low and middle income countries fail to protect the public from the economic consequences of dental care. Moving away from out-of-pocket payments to prepayment and risk-pooling mechanisms to protect households, at least for large health shocks, is likely to be beneficial for families and help rebalance the financial burden of health care costs [3]. A growing body of evidence from developing countries shows that increasing fairness in the distribution of health spending tends to improve both equity in the use of services and financial protection [33,34]. Policy makers could also consider the abovementioned determinants of CDHE in tailoring social protection policies for specific sub-groups of the population. There is an opportunity for dental public health advocates and international dental organizations to incorporate dental care in current discussions about universal health coverage and its role in achieving equity in the use of health services [35]. Future research should focus on three areas: first, the mechanisms families use to cope with out-of-pocket-payments; second, the specific role of health and dental health insurance in reducing CDHE; and third, what specific dental services may force families into catastrophic payments.

## Conclusions

This analysis of 41 low and middle income countries shows that payments for dental health care can put a considerable burden on households to the extent of preventing expenditure on basic necessities. The present findings also help characterize which households are more likely to incur catastrophic expenditure in dental health care. Our findings indicate the lack of public protection from the financial consequences of dental care. Alternative healthcare financing strategies and policies targeted to improve fairness in financial contribution (such as tax-based health financing systems or social health insurance schemes) are urgently required in low and middle income countries.
